# Determinants of pneumococcal vaccination dropout among children aged 12–23 months in Ethiopia: a secondary analysis from the 2019 mini demographic and health survey

**DOI:** 10.3389/fpubh.2024.1362900

**Published:** 2024-07-03

**Authors:** Ayenew Assefa, Teklehaimanot Kiros, Mulat Erkihun, Aynework Abebaw, Ayenew Berhan, Andargachew Almaw

**Affiliations:** ^1^Unit of Immunology, Department of Medical Laboratory Science, Debre Tabor University, Debre Tabor, Ethiopia; ^2^Unit of Medical Microbiology, Department of Medical Laboratory Science, Debre Tabor University, Debre Tabor, Ethiopia; ^3^Unit of Parasitology, Department of Medical Laboratory Science, Debre Tabor University, Debre Tabor, Ethiopia; ^4^Unit of Hematology, Department of Medical Laboratory Science, Debre Tabor University, Debre Tabor, Ethiopia

**Keywords:** vaccine, children, dropout, women, demographic and health survey

## Abstract

**Background:**

Vaccination is a cost-effective public health program that helps reduce significant morbidity and mortality in children under the age of five. Worldwide, the number of vaccine-preventable causes of child death has significantly decreased since the Expanded Program of Immunization (EPI) was introduced. However, for a variety of reasons, 23 million children did not have adequate access to vaccines in 2020. Therefore, this study aimed to evaluate the determinants of pneumonia conjugate vaccine (PCV) dropout among children aged 12–23 months in Ethiopia.

**Methods:**

The study analyzed cross-sectional data obtained from the 2019 mini Ethiopian demographic and health survey. Multilevel binary logistic regression analysis was utilized, and the best fit model was chosen using the Akaike Information Criteria. The study comprised a weighted sample of 989 children aged 12 to 23 months. The study presented the Adjusted Odds Ratio (AOR) along with a 95% Confidence Interval (CI) to identify the significant factors influencing PCV dropout.

**Results:**

The PCV dropout rate was reported at 20.2% in this study. In the multilevel analysis, possession of a health card (AOR = 0.076, 95% CI: 0.019, 0.04), vaccination for PCV 2 (AOR =0.002, 95% CI: 0.023, 0.263), and region 7 (AOR = 6.98, 95% CI: 10.1, 48.31) were significantly associated with children’s PCV dropout.

**Conclusion:**

Having a health card, having received the PCV 2 vaccinations, and region were significant predictors of PCV dropout. Consequently, health education on immunization for all mothers and region-specific, customized public health interventions are needed to reduce the vaccination dropout rate.

## Introduction

Pneumonia is an acute lower respiratory tract infection that damages the alveolar air space and lung tissue. For children under the age of five, pneumonia is the greatest cause of illness and mortality (1). Over 900,000 children died from pneumonia worldwide in 2016, making up around 16% of the 5.6 million deaths of children under five (2). With 50% of the global death rate for children under five caused by pneumonia, Sub-Saharan African nations carried the lion’s share of the burden. In sub-Saharan African nations, pneumonia is the leading cause of death, accounting for about 172 deaths per 1,000 live births (3, 4). In Ethiopia, pneumonia is the number one cause of death in the postnatal period as well as the primary cause of morbidity and mortality in children under the age of five. Each year, more than 40,000 children under the age of five die from pneumonia, which accounts for 20% of all causes of death (5, 6).

Pneumonia, which causes significant morbidity and mortality in children under the age of five, is among the diseases that can be prevented by vaccination. Vaccination is a cost-effective public health program that helps reduce these rates. Worldwide, the number of vaccine-preventable causes of child death has significantly decreased since the Expanded Program of Immunization (EPI) was introduced in 1974 with the goal of boosting routine immunization coverage (7). Around 2 to 3 million children a year are saved through vaccination (8). By the end of 2021, the pneumococcal vaccine had been launched in 154 member states, and global third-dose coverage was anticipated at 51% (9). In November 2011, Ethiopia added the 10-valent pneumococcal conjugate vaccine (PCV 10) to its childhood immunization schedule with the help of Gavi, the Vaccine Alliance. In accordance with the national vaccination program’s three-dose schedule, children receive the shot at 6, 10, and 14 weeks of age (10).

In accordance with World Health Organization (WHO) recommendations, children are deemed fully immunized when they have received the following vaccinations by the age of 12 months: BCG for tuberculosis, three doses of DPT-Hep B-Hib (diphtheria, pertussis, and tetanus), pneumonia-conjugate vaccine (PCV) and polio, two doses of Rota virus, and a measles shot (11). In 2011, the national immunization program added the three doses of PCV to the vaccination schedule (12).

According to Ethiopia’s routine vaccination schedule, infants should begin receiving vaccinations at birth and finish them before turning 1 year old. This includes receiving a single dose of the Bacillus Calmette-Guerin (BCG) vaccine at birth or as soon as possible, as well as the first dose of the oral polio vaccine (OPV). Three doses of the OPV, Pentavalent, Rota1, Rota 2, and pneumonia vaccines are given at intervals of 4 weeks duration at the 6^th^, 10^th^, and 14^th^ weeks, respectively, and finally, the measles vaccine is given at the age of 9 months (13) (Table 1). 90% national coverage and 80% district coverage goals were set by the Global Vaccine Action Plan and the EPI for the year 2020 (14).

**Table 1:** Routine immunization schedule in Ethiopia.

Vaccine	Disease	Age
BCG	Tuberculosis	At birth
Pentavalent	Diphtheria, Pertussis, Tetanus, H. influenza type b, Hepatitis B	6, 10, 14 weeks
OPV	Polio	At Birth, 6, 10, 14 weeks
Measles	Measles	9 Months
Pneumonia-conjugate Vaccine (PCV)	Pneumonia	6, 10, 14 weeks
Rotarix (rotavirus vaccine)	Rotavirus	6, 10 weeks
Tetanus (TT) immunization for women in child bearing age	Tetanus	1st contact pregnancy; +1 month, +6 months; +1-year, +1 year

Even though immunization rates had increased, in 2020, some 23 million children still lacked sufficient access to the shot (15–17). By the end of 2020, 83% fewer children worldwide have received their childhood vaccinations than there were in 2019 (18). Around 60% of these children resided in low-and middle-income nations (19). According to data from an Ethiopian demographic health survey, 39% of children aged between 12 and 23 months in 2016 received all required vaccinations. Because of this, the country’s immunization rates are often below the threshold needed to create herd immunity and stop the spread of eight EPI-targeted diseases (20). Numerous studies have shown that factors such as home birth, residence, mother’s knowledge of immunization, home visits by health workers, distance to medical facilities, misunderstandings about the benefits of immunization, and lack of knowledge about vaccine contraindications were predictors for child immunization (21–23).

Full immunization coverage in Ethiopia remained extremely low, at 33.3%, for all age-appropriate immunizations, including three doses of PCV, despite the government’s reform initiatives. In addition, there is a notable variation in immunization coverage across various parts of the country (24). Several studies have examined the numerous factors that may contribute to childhood immunization dropouts (25). To our knowledge, few studies have been done on the rates of measles and polio coverage, but there have not been any studies on PCV coverage or dropout rates using survey data from Ethiopia. Therefore, this study sought to evaluate the determinants of the PCV dropout rate in Ethiopia using the 2019 mini-DHS data.

## Methods and materials

### Data source and study subjects

The 2019 Ethiopian mini demographic and health survey (EMDHS) data served as the data source for the analysis. It is the second EMDHS and the fifth DHS implemented in Ethiopia. The survey was conducted by the Ethiopian Public Health Institute (EPHI) in collaboration with the Central Statistical Agency (CSA), the Federal Ministry of Health (FMoH), financial and technical support from development partners, and technical assistance from the Inner City Fund (ICF). The survey was conducted from March 21, 2019 to June 28, 2019, based on a nationally representative sample that provided estimates at the national and regional levels and for urban and rural areas.

Two administrative cities (Addis Ababa and Dire Dawa) and all nine regions of Ethiopia (Tigray, Afar, Amhara, Benishangul-Gumuz, Gambela, Harari, Oromia, Somalia, and the Southern Nations, Nationalities, and People’s Region, or SNNP), were included in the nationally representative sample (26). The two administrative cities and the nine regions were grouped into three categories in our analysis: city administrations (Addis Ababa and Dire Dawa), developed regions (Tigray, Amhara, Oromia, and SNNP), and emerging regions (Afar, Benishangul-Gumuz, Gambela, Harari, and Somalia). 8,855 reproductive-age women (ages 15 to 49) from a nationally representative sample of 8,663 households were interviewed for the survey. All census enumeration areas (EAs) established for the 2019 Ethiopia Population and Housing Census (EPHC), which was carried out by the CSA, served as the sampling frame for the 2019 EMDHS. All 149,093 EAs developed for the 2019 EPHC are included in the census frame.

The 2019 EMDHS sample was stratified and selected in two stages. 21 sampling strata were produced by stratifying each region into urban and rural areas. EA samples were chosen in two stages, independently, for each stratum. In the first stage, a total of 305 EAs (93 in urban areas and 212 in rural areas) were selected with probability proportional to EA size and with independent selection in each sampling stratum. A household listing operation was carried out in all selected EAs, and the resulting lists of households served as a sampling frame for the selection of households in the second stage.

In the second stage of selection, a fixed number of 30 households per cluster were selected with an equal probability of systematic selection from the newly created household listing. All women age 15–49 who were either permanent residents of the selected households or visitors who slept in the household the night before the survey were eligible to be interviewed. The study included children between the ages of 12 and 23 months who did have complete data on their age and outcome variable. Those children with missing data in the DHS dataset were excluded, and the final analysis comprised a sample of 989 children aged 12 to 23 months.

The 2019 EMDHS was used to extract pertinent data regarding vaccinations for children aged 12 to 23 months. This was completed after registering and submitting the study’s proposal via the website to obtain ICF international approval for access to and use of the dataset. After receiving authorization via email, the dataset was downloaded from the http://www.DHSprogram.com website.

### Outcome variable

PCV dropout for children between the ages of 12 and 23 months was the study’s outcome variable. Vaccination dropout refers to a child who has received the first dose of a vaccination according to recommendation but has missed the next dose (the third dose). A child who received the first dose of the PCV vaccination and not the last (third) was classified as “dropping out of PCV” and labeled “yes,” while a child who received the first and last doses was classified as “no.” The dropout rate was calculated by subtracting the frequency of PCV 1 status from PCV 3 status and dividing it by 100.

### Independent variables

Based on various works of literature, the potential variables associated with childhood immunization dropout were divided into two categories: individual-and community-level variables. The variables at the individual level include the mother’s age, mother’s educational status, marital status, wealth index, number of antenatal care (ANC) visits, place of delivery, presence of a health card, child sex, birth order, number of children ever born, and vaccination received for PCV2. Community level variables were urban–rural status, region, and aggregated variables created from individual level variables including community wealth index, community women’s education, and community place of residence. Since the EMDHS data were not normally distributed, aggregated community variables were classified as low or high using the median value (27, 28).

### Statistical analysis

IBM SPSS statistical software version 25 was used for the analysis. Weighting was utilized to correct for the sample’s non-proportional distribution among the various regions and their rural and urban areas. Thus, it is ensured that the survey results are representative at the national and regional levels. The variables were described using percentages and frequencies, and tables and a figure were used to display the data.

Women and children were nested within a cluster in the DHS data; these clusters may have shared characteristics. The conventional logistic regression model’s assumptions of equal variance and independence of observations are broken by the hierarchical structure of the data. Consequently, the clustering effect was taken into consideration by fitting a multilevel logistic regression model (both random and fixed effects). The community-and individual-level variables associated with PCV dropout were found using the two-level mixed-effects logistic regression model. In this study, four successive models were fitted. The first model is called the null model, and it does not contain any variables, whereas Model I has only individual level variables. Only community level variables are used to build Model II, and both individual and community level variables are combined to create Model III, a mixed model.

A two-level mixed model with individuals nested within communities was fitted. In order to model the log odds of children receiving a full immunization (29):


Yij=Y00+yp0Xpij+y0qZqj+u0j

Where γ00 represents the intercept—that is, the probability of immunization in the absence of explanatory variables—and Yij represents the full immunization status of the i^th^ child in the j^th^ cluster. The regression coefficient of the individual level variable, Xp, is denoted by the term γp0, while the regression coefficient of the community level variable, Zq, is represented by γ0q. The explanatory variables at the individual and community levels are Xp and Zq, respectively. The community level error is denoted by the term u0j, where the subscripts i and j stand for the individual level and cluster number, respectively.

The best fit model was determined by calculating the model’s lowest Akaike Information Criteria (AIC), and the best-fitting model was determined to be the mixed model (30). This model demonstrated that both individual and community level variables account for 49.3% of the total variance in the odds of children dropping out of PCV vaccination. For the multivariable multilevel analysis, variables from the bivariable multilevel analysis with a *p* value <0.2 were considered. A *p*-value of 0.05 was established as the cut point for statistical significance. The measures of association between the odds of PCV dropout and the various explanatory variables were expressed as Adjusted Odds Ratios (AOR) with their 95% CIs (Table 2).

**Table 2:** Estimates of the random effects of community and individual level factors on children’s PCV dropout rate in Ethiopia in 2019.

Estimates	Null model	Model I	Model II	Model III
AIC	4888.73	1314.25	4015.85	1011.01
PCV (%)	Reference	46.6	41.3	49.3
ICC (%)	43	28.7	30.7	27.7
Community level variance	2.482*	1.326	1.458	1.259^*^

*p value < 0.001, PCV = proportional change in variance.

By calculating the Intra-class Correlation Coefficient (ICC), the null model was fitted in order to estimate the clustering effect and support the use of multilevel analysis. The formula below was used to determine ICC-the percentage of variability explained by the upper level (community) (30). ICC = $\frac{VA}{VA+\pi 2/3\;}$ , where VA is community level variance and $\frac{\pi 2}{3}$ is individual level variance, equals 3.29. Consequently, community differences account for roughly 43% of the overall variation in the odds of children experiencing PCV dropout. ICC = 0.05 is often regarded as a conventional threshold to indicate more substantial evidence of clustering (31). In the null model, the clustering effect-related variance was 43; in models I, II, and III, it was 28.7, 30.7, and 27.7, the lowest in the mixed model. The proportional Change in Variance (PCV) was computed with respect to the null model in order to investigate the relative contributions of individual and community level variables in explaining children’s PCV dropout. Proportional Change in Variance (PCV) = $\frac{V0-Vi}{V0}$ , where variance in the null model is represented by Vo, and variance in the subsequent models is represented by Vi (30). Model III exhibits the highest proportional change in variance, indicating that the combination of individual and community level variables accounts for a larger portion of the variation in children’s PCV dropout rate than either one does on its own.

### Ethical considerations

The 2019 EMDHS was conducted by the Ethiopian Public Health Institute (EPHI) in collaboration with the Federal Ministry of Health (FMoH), financial and technical support from development partners, and technical assistance from the Inner-City Fund (ICF). Before every interview during the survey’s implementation, participants were asked to provide their informed consent. The project proposal’s concept note and title were submitted via the DHS website in order to register and obtain authorization to use the EDHS data. The EDHS data set was not disclosed to a third party without first requiring registration, and the accessed data was only used for this study.

## Results

### Dropout rate

**Figure 1:**
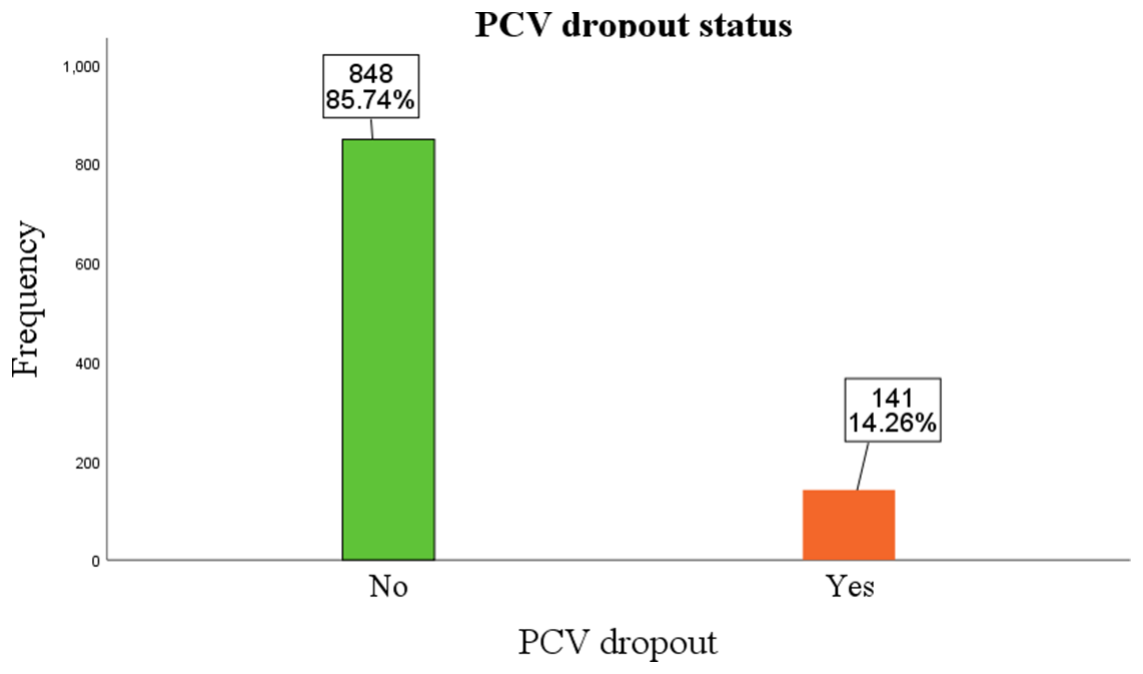
Percentage of children who drop out of PCV. Children categorized as “Yes” were those who dropped out, and those in the “No” category were not dropped out of the vaccination. Accordingly, 141 (14.3%) of the children dropped out of the PCV vaccination, while 848 (85.7%) of them were not.

The dropout rate of PCV was reported at 20.2% in Ethiopia. In this study, 14.3% of the children were dropped out of PCV, compared to the majority of the children (85.7%) who were not (displayed in Figure 1).

### Individual level characteristics of the study subjects

A total of 989 children aged 12–23 months were included in the analysis. More than half of the respondents (52.9%) were in the age group between 25 and 34 years. 499 (50.5%) of the children were male. Majority (36.9%) of the children were found in 2–3 birth order, while 20.3, 20.3, and 19.8% of them were found in 1, 3–4, and 6–15 birth order, respectively. The majority (94%) of the respondents were married. The majority of mothers (48.1%) do not have a formal education. The households with the lowest wealth index comprised 46.1% of the mothers. 53.6% of the respondents, or more than half, have had four ANC visits. Children’s health cards were possessed by 72.2% of the mothers. Of the children in the study, males made up nearly half (50.5%). More than half (53.3%) of the children were born at home. In this study, 70.5 and 64.8% of children received the PCV 1 and PCV 2 vaccinations, respectively. On the other hand, 43.8% of children drop out of the PCV 3 vaccination (Table 3).

**Table 3:** Individual level characteristics of children 12–23 months of age in Ethiopia, 2019 (*n* = 989).

Characteristics	Frequency (n)	Percent (%)
Sex of child		
Female	490	49.5
Male	499	50.5
Birth order		
1	227	23.0
2–3	365	36.9
4–5	201	20.3
6–15	196	19.8
Mother’s age		
15–24	292	29.5
25–34	523	52.9
35–49	174	17.6
Marital status		
Married	930	94.0
Not married	59	6.0
Wealth index		
Poor	456	46.1
Middle	133	13.4
Rich	400	40.4
Educational level		
No education	476	48.1
Primary	341	34.5
Secondary and higher	172	17.4
Number of ANC visit		
0	138	28.9
1–4	256	53.6
5–20	84	17.6
Place of delivery		
Home	256	53.3
Health facility	224	46.7
Health card		
Yes	714	72.2
No	275	27.8
Received PCV 1		
Yes	697	70.5
No	292	29.5
Received PCV 2		
Yes	641	64.8
No	348	35.2
Received PCV 3		
Yes	556	56.2
No	433	43.8
Child live with		
Respondent	978	98.9
Elsewhere	11	1.1
Total of children ever born		
1–3	589	59.6
4–6	272	27.5
7+	128	12.9

### Community level characteristics of the study subjects

The majority of respondents (43.1%) came from developed regions (Amhara, Oromia, SNNPR, and Tigray), whereas the remaining 42.6 and 14.4% came from emerging regions and city administrations, respectively. Rural residents made up 73.2% of the respondents. More than two-thirds (67.4%) of mothers came from communities with a high percentage of poverty. The majority (57.1%) of the mothers were from communities with a high proportion of deliveries at home. About seven out of 10 (69.2%) of the participants came from areas where the percentage of uneducated women was high (Table 4).

**Table 4:** Community level characteristics of children 12–23 months of age in Ethiopia, 2019 (*n* = 989).

Variables	Frequency (N)	Percentage (%)
Urban–rural status		
Urban	265	26.8
Rural	724	73.2
Region		
Emerging regions	426	43.1
Developed regions	421	42.6
City administrations	142	14.4
Community women education		
Low	684	69.2
High	305	30.8
Community wealth index		
Low	322	32.6
High	667	67.4
Community place of delivery		
Low	424	42.9
High	565	57.1

### Significant predictors of PCV dropout

In our study, the presence of a health card and PCV 2 vaccination status were significant individual level factors that affected children’s PCV dropout rates, whereas region was a statistically significant community level factor. Children of respondents who have a health card were 92.4% (AOR = 0.076, 95% CI: 0.019, 0.04) less likely to drop out of PCV than those who have no health card. Likewise, children who received PCV 2 were 99.8% (AOR =0.002, 95% CI: 0.023, 0.263) less likely to drop out of PCV vaccination than those who did not received it. Respondents from emerging regions were 7 (AOR = 6.98, 95% CI: 10.1, 48.31) times more likely to drop out of PCV vaccination than city administrations (Table 5).

**Table 5:** Individual and community level factors associated with PCV dropout of children 12–23 months of age in Ethiopia, 2019 (*n* = 989).

Variables	Vaccination status frequency (%)		AOR (95% CI)
	Yes	No	
Health card			
Yes	104 (14.6)	610 (85.4)	0.076 (0.019, 0.309)^***^
No	37 (13.5)	238 (86.5)	1
Residence			
Urban	27 (10.2)	238 (89.8)	0.22 (0.003,3.796)
Rural	114 (15.7)	610 (84.3)	1
Sex of child			
Male	72 (14.4)	427 (85.6)	1
Female	69 (14.1)	421 (85.9)	0.45 (0.203, 2.301)
ANC visit			
Yes	29 (21.0)	109 (79.0)	0.18 (0.608, 14.208)
No	52 (15.2)	290 (84.8)	1
Wealth			
Poor	74 (16.2)	382 (83.8)	1
Middle	18 (13.5)	115 (86.5)	0.44 (0.62, 3.376)
Rich	49 (12.3)	351 (87.8)	0.69 (0.327, 5.461)
Received PCV 2			
Yes	85 (13.3)	556 (86.7)	0.002 (0.023, 0.263)^**^
No	56 (16.1)	292 (83.9)	1
Regions			
Emerging regions	60 (14.1)	366 (85.9)	1
Developed regions	62 (14.7)	359 (85.3)	4.06 (1.54, 10.71)
City administrations	19 (13.4)	123 (86.6)	6.98 (10.1, 48.31)^*^

^*^p < 0.05, ^**^p < 0.02, ^***^p < 0.001.

## Discussion

Dropout rates are frequently used as a gauge of how well immunization programs are working, and low dropout rates signify good access to and use of immunization services (32). This study aimed to concurrently uncover the community-level and individual-level factors influencing children’s PCV dropout rate in Ethiopia. Thus, the individual level factors influencing PCV dropout were having a health card and receiving a PCV 2 vaccination. One community-level factor influencing children’s PCV dropout rate was region.

The PCV dropout rate was found to be 20.2% in the current study. Therefore, intervention is necessary as a dropout rate in the EPI program of more than 10%, reported by the WHO and the Global Health Survey (GHS), calls for action (33). There are a number of speculations as to why vaccination dropout rates are higher, including forgotten dose reminders, lack of vaccines, rejection because a card is misplaced, absence of counseling, caretakers’ fear of vaccine side effects, absence of postpartum care visits, and financial difficulties with transportation (11, 34). It has been demonstrated that strategically placing sticker reminders with suggested vaccination return dates throughout the home can lower the dropout rate (35).

This study demonstrated a significant association between children’s PCV dropout and the possession of a health card. A health card is a vaccination record that the parents of the children (mothers), own. It lists all of the vaccines that the child has received and the dates of those shots. It also includes details such as the child’s age and the vaccine schedule’s lot number for each dose of a vaccine. In addition, the vaccination card can serve as a forecast, informing parents of when their child should see the vaccination provider again to finish a series of shots or begin a new one (36). Children of mothers who possess a health card have a lower rate of PCV dropout compared to children of mothers who do not own a health card. Parallel to our findings, several studies from Ethiopia (28, 37), Ghana (38), Gambia (25), Nigeria (39), and East China (40) showed that a health card is a significant determinant factor for children’s vaccination status. This might be because mothers will find it simple to remember their child’s appointment if they own a health card, and health providers will find it easy to inform and monitor the status of vaccinations. Identifying and counseling mothers who have vaccination cards may also be a simple task for health extension workers during house-to-house visits (25, 41).

One community-level factor that was significantly associated with the PCV dropout rate of children was region. The odds of PCV dropout were higher in children of respondents who reside in emerging regions as compared to those who live in city administrations. In line with what we found, a number of studies showed that region has a significant association with children’s vaccination status (25, 27, 28, 42, 43). This could be because most respondents from emerging regions are from remote, hard-to-reach areas, and mothers there are less aware of the advantages of receiving all recommended vaccinations, EPI sessions, and health services. As opposed to this, respondents living in city administrations may have greater access to media outlets like radio, television, and newspapers, which raises their likelihood of being aware of vaccinations. Moreover, the availability of health services, including the immunization program, varies by region, which could be another explanation for this variation (27, 42, 44).

Vaccination against PCV 2 was also significantly associated with children’s PCV dropout. The odds of PCV dropout were lower among children who received PCV 2 vaccination. This could be because, in the aftermath of PCV 2, mothers may have been confident enough to proceed with the vaccination due to adequate information. Furthermore, improved immunization campaigns may have made mothers aware of the advantages of receiving all doses.

### Limitations

The study has limitations in addition to its merits. Causal and temporal inferences cannot be made because the study used a cross-sectional study design. The men’s variable and TT vaccination for women were missing in the 2019 mini EDHS data and not included in the analysis.

## Conclusion

In this study, the PCV dropout rate was reported to be 20.2% among children aged 12–23 in Ethiopia. Moreover, having a health card and having received the PCV 2 vaccination were individual level significant predictors of PCV dropout, and one community-level factor that was significantly associated with PCV dropout was region. Consequently, health education on immunization for all mothers and region-specific, customized public health interventions are needed to reduce the PCV vaccination dropout rate. Furthermore, since children are more likely to miss their vaccinations if their mothers do not have a health card, it is critical to counsel or raise mothers’ awareness of the importance of a health card. An Android system that automatically reminds health providers and mothers, if appropriate, is also helpful to minimize vaccination dropout, even though it requires further research.

## Data availability statement

The original contributions presented in the study are included in the article/supplementary materials, further inquiries can be directed to the corresponding author.

## Ethics statement

Ethical approval was not required for the study involving humans in accordance with the local legislation and institutional requirements. Written informed consent to participate in this study was not required from the participants or the participants' legal guardians/next of kin in accordance with the national legislation and the institutional requirements.

## Author contributions

AAs: Conceptualization, Supervision, Writing – original draft, Writing – review & editing. TK: Formal analysis, Software, Writing – review & editing. ME: Formal analysis, Writing – review & editing. AAb: Formal analysis, Writing – review & editing. AB: Formal analysis, Writing – review & editing. AAl: Formal analysis, Writing – review & editing.
